# Influences of Dominance and Evolution of Sex in Finite Diploid Populations

**DOI:** 10.1371/journal.pone.0128459

**Published:** 2015-05-26

**Authors:** Yujun Chang, Yuan Hua, Xiaoqian Jiang, Shiheng Tao

**Affiliations:** 1 State Key Laboratory of Crop Stress Biology for Arid Areas, Bioinformatics Center, College of Life Sciences, Northwest A&F University, Yangling, Shaanxi, China; 2 Department of Biology, Indiana University, Bloomington, Indiana, United States of America; CNR, ITALY

## Abstract

Most eukaryotes reproduce sexually. Although the benefits of sex in diploids mainly stem from recombination and segregation, the relative effects of recombination and segregation are relatively less known. In this study, we adopt an infinite loci model to illustrate how dominance coefficient of mutations affects the above-mentioned genetic events. However, we assume mutational effects to be independent and also ignore the effects of epistasis within loci. Our simulations show that with different levels of dominance, segregation and recombination may play different roles. In particular, recombination more commonly has a major impact on the evolution of sex when deleterious mutations are partially recessive. In contrast, when deleterious mutations are dominant, segregation becomes more important than recombination, a finding that is consistent with previous studies stating that segregation, rather than recombination, is more likely to drive the evolution of sex. Moreover, beneficial mutations alone remarkably increases the effects of recombination. We also note that populations favor sexual reproduction when deleterious mutations become more dominant or beneficial mutations become more recessive. Overall, these results illustrate that the existence of dominance is an important mechanism that affects the evolution of sex.

## Introduction

Sexual reproduction is ubiquitous among eukaryotes in nature. However, the evolution of sex is difficult to explain owing to the cost of sex such as an excess of energy expenditure is involved in finding willing mates for sex [1, 2]. Among the numerous explanations about sex, a well-known hypothesis claims that the presence of sex influences genetic variation. In haploids, the advantage of sex or the genetic variation is primarily due to recombination; whereas in diploids, segregation also influences the genetic variation [3, 4]. It is well known that recombination breaks down associations between different loci, and segregation breaks down associations within the same locus [5, 6]. This fact indicates that haploid models are not suitable to explain the evolution of sex in diploids.

Recombination aspects have been explored in diploids under various conditions [7]. Most of these theoretical models involved computer simulations, wherein diploid populations reproduced with obligate mutations. For instance, recessive deleterious mutations tend to select against recombination [8], whereas the presence of beneficial mutations results in the higher fixation rate of recombination modifiers [9]. Genetic drift generates negative linkage disequilibrium and thus favors the development of higher recombination rates [10]. Other factors can also affect selection on recombination, such as population structure, interference among mutations, and host–parasite interactions [11–14].

Using computer simulations, previous studies also focused on the benefits of segregation. Under the assumption of host–parasite coevolution in diploids, researchers modeled the interaction between a single host species and a single parasite species and suggested that the advantages of segregation might be more generalized explanations for the evolution of sex [15, 16]. In the presence of segregation, inbreeding could also provide an advantage to sexual reproduction by creating an excess of homozygotes [17]. On the basis of the above-mentioned theoretical studies, segregation has been found to be very important for the evolution of sex.

A few other studies have also focused on the comparison between the effects of recombination and segregation. For instance, Agrawal (2009) compared their relative effects by using mutation–selection balance model with three loci [17] and demonstrated that as an important factor, migration between genetically differentiated populations could impact the evolution of sex by influencing segregation. In a previous study, we also showed that in multi–locus models, the combined effects of segregation and recombination strongly contributed to the evolution of sex in diploids [18]. However, another critical determinant, the dominance coefficient, *h*, was not considered in that study. In the present study, we focused on how dominance would really impact the relative effects of these genetic events in diploids.

The dominance coefficient measures the fitness of interactions between mutations within the same locus, and evidently, it has a lasting influence on the evolutionary process [2]. The fixation of new mutations is also closely related to the dominance coefficient. The principle termed Haldane’s sieve posits that most advantageous mutations that are fixed in the population should be dominant rather than recessive [19]. But how Haldane’s sieve works with different dominance coefficients has not yet been studied. The fixation of deleterious mutations will be retarded when the dominance coefficient is sufficiently less, as found by Charlesworth and Charlesworth (1997). Nevertheless, the mechanism by which dominance coefficient influences the fixation of beneficial mutations remains elusive. Meanwhile, populations disfavor recombination when the dominance coefficient of deleterious mutations is sufficiently small [8, 20]. However, it is not yet clear how dominance influences the relative effects of recombination and segregation in finite diploids. In the present study, computer simulations were employed to explore the effects of dominance with the assumption of two scenarios. First, we investigated the effects of dominance through a direct comparison of asexual and sexual populations after thousands of generations evolved. Second, we utilized a sex modifier model to explore the effects of dominance on the evolution of sex. Both deleterious and beneficial mutations were considered in our simulations, and the results suggested that segregation more commonly has a major impact when deleterious mutations were partially dominant or when beneficial mutations were partially recessive. Meanwhile, segregation was more likely to drive the evolution of sex under the same conditions.

## Methods

### General settings

Simulation programs were written in C++ with reference to Roze (2006). In our simulation, each population consisted of *N* mutation-free diploid individuals and the genome of each individual contained only one pair of chromosomes. We assumed non-overlapping generations that included mutations and reproduction processes (incorporating segregation and recombination). Offspring were produced according to their fitness, wherein the fitness was weighted by all genotypes of whole alleles. Considering that a locus contained alleles *A* and *a*, if the selection coefficient of deleterious mutation in *a* was *s*_*D*_, then the fitness *w* of three different genotypes *AA*, *Aa* and, *aa* could be written as:

$$w_{AA}=1,\;w_{Aa}=1-hs_{D},\;w_{aa}=1-s_{D}.$$ (1)

When *a* referred to beneficial mutations with selection coefficient *s*_*B*_, Eq (1) could be rewritten as:

$$w_{AA}=1,\;w_{Aa}=1+hs_{B},\;w_{aa}=1+s_{B},$$ (2)

where *h* stood for the dominance coefficient of the mutation. The minimum value of *h* was set to 0.1, as estimates in *Drosophila melanogaster* and *Saccharomyces cerevisiae* indicated that *h* > 0.01 [21, 22]. The mutation in allele *a* was partially recessive if dominance coefficient *h* < 0.5, whereas it was partially dominant when *h* > 0.5. According to observations in eukaryotes, *s*_*B*_ and *s*_*D*_ were set at 0.02 and 0.05 respectively [23–25].

### Description of reproduction process

Each diploid individual contained two chromosomes with infinite loci in our simulation. Every new mutation occurred at a unique position defined by a random value between 0 and 1.0. These values were sampled from a standard uniform distribution. The number of mutations occurring per generation for each individual was sampled from a Poisson distribution with parameter *U*_*D*_ (or *U*_*B*_). Both deleterious and beneficial mutations were considered here, where *U*_*D*_ represents the mutation rate of deleterious mutations whose value was set to 0.2, and *U*_*B*_ stands for the mutation rate of beneficial mutations with value set to 0.002. The value of *U*_*D*_ was chosen according to the estimated range of deleterious mutation rates in *D*. *melanogaster* [26–28]. Considering different mutation scenarios helped explore the relative effects of recombination and segregation clearly.

The probability of an individual reproducing offspring was determined by its fitness. The fitness *w*_*i*_ of an individual *i* was calculated as:

$$w_{i}=w_{aa}{}^{N_{ho}}\;w_{Aa}{}^{N_{he}},$$ (3)

where *N*_*ho*_ and *N*_*he*_ were the number of homozygous and heterozygous mutations that an individual carried, respectively. *L* stood for the linear genetic map length per chromosome (*L* ≥ 0). The number of recombination events that occurred between homologous chromosomes was sampled from a Poisson distribution using a mean value of *L*, and the recombination events were uniformly distributed among the chromosomes.

### Simulation of fully asexual/sexual populations

In this part of the simulation, three different populations were considered: asexual population, sexual population without recombination (S population), and sexual population with recombination (S&R population). Meanwhile, two different mutation scenarios, mutations were exclusively deleterious and exclusively beneficial, were considered independently. For an S&R population based on weak recombination, the value of *L* was kept constant to 0.01 M. However, for an S population, there was no recombination in the reproduction process and the value of *L* was 0 M. The initial fitness of each individual was uniformly set to 1.0 in the simulation.

In a fully asexual population, each asexual individual was produced as follows. Before the process of reproduction, an individual randomly chosen from a previous generation would be considered as the parent of a new offspring only if *R* < *w*_*i*_/*w*_*max*_, where *w*_*i*_ was the fitness of this individual, *w*_*max*_ was the maximum fitness in this population. Different *R* was sampled from a standard uniform distribution when each new individual chosen from previous generation. The purpose of this step was to make sure that an individual with higher fitness value was more likely to generate an offspring in our random mating system. The choosing procedure was same in all of the three populations. For the fully sexual population, two individuals were successively chosen from previous generation as parents of a new offspring before the mating process. Then a new offspring was produced by combining two chromosomes from the parents separately. In S&R population, recombination was introduced based on the S population by chromosomal crossover between the paired chromosomes inherited from each of the parents. The difference among the three populations was whether recombination and segregation occurred or not: both genetic events did not occur in the asexual population; only segregation occurred in the S population; and both events occurred in the S&R population. Thus, the relative effects of segregation could be assessed by comparing these three populations.

All the populations reproduced for a certain number of generations. The mean fitness of each population (*W*), the number of fixed deleterious mutations (*N*_*D*_) or beneficial mutations (*N*_*B*_), and the genetic diversity (*V*_*g*_) were recorded until the population evolved 5000 generations. Each parameter combination was run 10 times and the standard error was calculated. We did not consider different selection coefficients, because various selective strength had been studied previously by our group [18]. This research concentrated on the influence of dominance with constant selective coefficients. Besides, both epitasis and back mutations were excluded from the simulation.

### Simulation of a sex modifier

The simulation of a sex modifier is different from that of the fully asexual or sexual population. First, the asexual population reproduced 2500 generations independently. During this burn-in period, all individuals produced fully asexual offspring and the population was generally sufficient to approach a dynamic balance. Second, we introduced only one sex modifier to a randomly chosen individual in this population. As a sexual individual (individual with a sex modifier) needs another sexual partner to produce offspring, we allowed the first individual carrying a sex modifier to reproduce asexually. When two or more sexual individuals appeared in this population through selection or genetic drift, these sexual individuals could produce offspring by sexual reproduction. During the reproduction process, an individual was chosen from a previous generation according to its fitness at first. If an individual without sex modifier was chosen, it would produce an asexual offspring. If an individual with a sex modifier was chosen, another sexual individual would be selected in the same way as its sexual partner. A sexual offspring was reproduced from the sexual parents. In order to create *N* offspring and keep the population size constant, we repeated this reproduction process *N* times.

We calculated the number of sex modifiers in each generation until it was fixed or lost in the population. For each burn-in population, we repeated the process, introducing single sex modifier and tracking the fate of sex modifier, *N* times to estimate the fixation probability of sex modifier *u*. The relative fixation probability of the sex modifier *u/u* ^*^ was calculated to disclose the relative advantage of sexual reproduction, where *u* ^*^ means the fixation probability of a neutral mutation. For each parameter combination, the whole simulation process was repeated 10 times to estimate the average value of *u/u* ^*^.

### Measuring genetic diversity among populations

To estimate the genetic diversity among the populations, we calculated the genetic diversity (*V*_*g*_) of each population. As fixed mutations did not contribute to selection in the reproductive process for a population, a “garbage collection” was executed every 200 generations to remove fixed mutations and accelerate the execution of simulations [9]. The genetic diversity (*V*_*g*_) was contributed by the number of alleles and could be computed using the following equation [8, 29]:

$$V_{g}=\sum_{i=1}^{m}p_{i}q_{i}$$ (4)

Here, *p*_*i*_ and *q*_*i*_ are the respective frequencies of the alleles *A* and *a* at locus *i* of the population, and *m* represents the number of overall mutated loci in the population.

## Results

### Mean fitness of population

**Fig 1:**
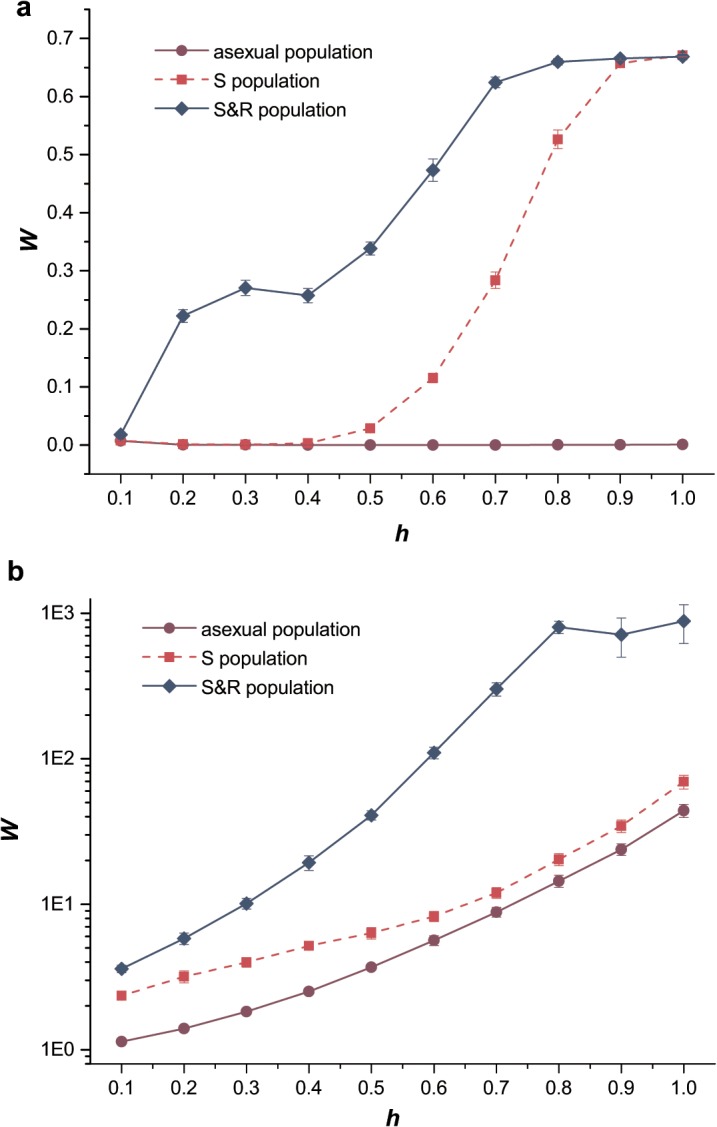
The mean fitness of populations. Purple circles, solid lines, asexual population; red squares, dashed lines, sexual population with only segregation; blue diamonds, solid lines, sexual population with both segregation and recombination (*L* = 0.01). (a) The mean fitness, *W*, of each population as a function of dominance coefficient of deleterious mutations *h* when mutations were exclusively deleterious. *U*_*D*_ = 0.2, *s*_*D*_ = 0.05. (b) The logarithm *W* as a function of *h* when mutations were exclusively beneficial. *U*_*B*_ = 0.002, *s*_*B*_ = 0.02. All the populations have the same size *N* = 10,000. Error bars are the standard error over the 10 averages here and throughout the article.

To assess the effects of dominance on segregation and recombination, we should evaluate the effects of dominance on mean fitness, and so we calculated the mean fitness (*W*) of each population in the simulation and recorded the balanced value of *W* after 5000 generations. Different dominance coefficients of mutations *h* were considered in two scenarios, exclusively deleterious mutations and exclusively beneficial mutations. We compared the relative effects of recombination and segregation on *W* (Fig 1). The asexual population had obviously lower mean fitness than sexual populations (S population and S&R population) in both scenarios. The lower *W* of asexual population was caused by the continuous accumulation of deleterious mutations in the heterozygous state, this process was known as Muller’s ratchet [30–32]. However, in asexual population, the higher value of *h* could slow down the rate of Muller’s ratchet (S1 Fig), which performed similarly with selection coefficient and had been confirmed in asexual populations [33, 34]. However, in the sexual population, segregation in S population eliminated parts of deleterious mutations and suspended Muller’s ratchet. The interaction between segregation and recombination in S&R population removed the deleterious mutations more efficiently and obtained a higher *W*, which meant the termination of Muller’s ratchet.

When deleterious mutations were partially dominant (*h* > 0.5), the increasing extent of *W* from asexual population to sexual population (S population) was higher than the case when *h* < 0.5. In a similar way, this increment of log_10_ (*W*) from asexual population to sexual population was also higher for beneficial mutations when *h* < 0.5. For a population with allele *A* and the mutant allele *a*, segregation could balance the distribution of alleles by converting intermediate genotypes to extreme genotypes (*Aa × Aa → AA*, *Aa*, *aa*) or vice versa. When the newly arising mutation *a* was deleterious and partially dominant (or beneficial and partially recessive),

$$w_{Aa}<\frac{1}{2}\left(w_{AA}+w_{aa}\right),$$ (5)

the average fitness of these two extreme genotypes was greater than that of the intermediate genotype. Therefore, segregation balanced the mutation of *Aa* to *AA* and *aa* by mating *Aa × Aa* under sexual reproduction, which, therefore, decreased the excess of heterozygosity and increased the effectiveness of selection, resulting in an increased *W*. In cases where mutations were deleterious and partially dominant, or beneficial and partially recessive, segregation was shown to be the major factor that increased the mean fitness of populations. The occurrence of recombination in sexual population (S&R population) broke the linkage disequilibrium generated by the Hill–Robertson effect, which meant that random linkage disequilibria would tend to slow down the process of evolution, and, therefore, increased *W* in all cases [35, 36]. All these results confirmed that *h* = 0.5 is an important dividing line in sexual reproduction affecting the relative strength of segregation and recombination.

Moreover, *W* increased exponentially in the presence of recombination when *h* increased from 0.1 to 1.0 by breaking the Hill–Robertson interference. This indicated that recombination was a major factor that affects the population with beneficial mutations. This result is also consistent with a previous conclusion stating that the presence of beneficial mutations leads to substantial selection on increasing the recombination rate [9].

### Influence of *h* on *N*_*D*_, *N*_*B*_, and *V*_*g*_

**Fig 2:**
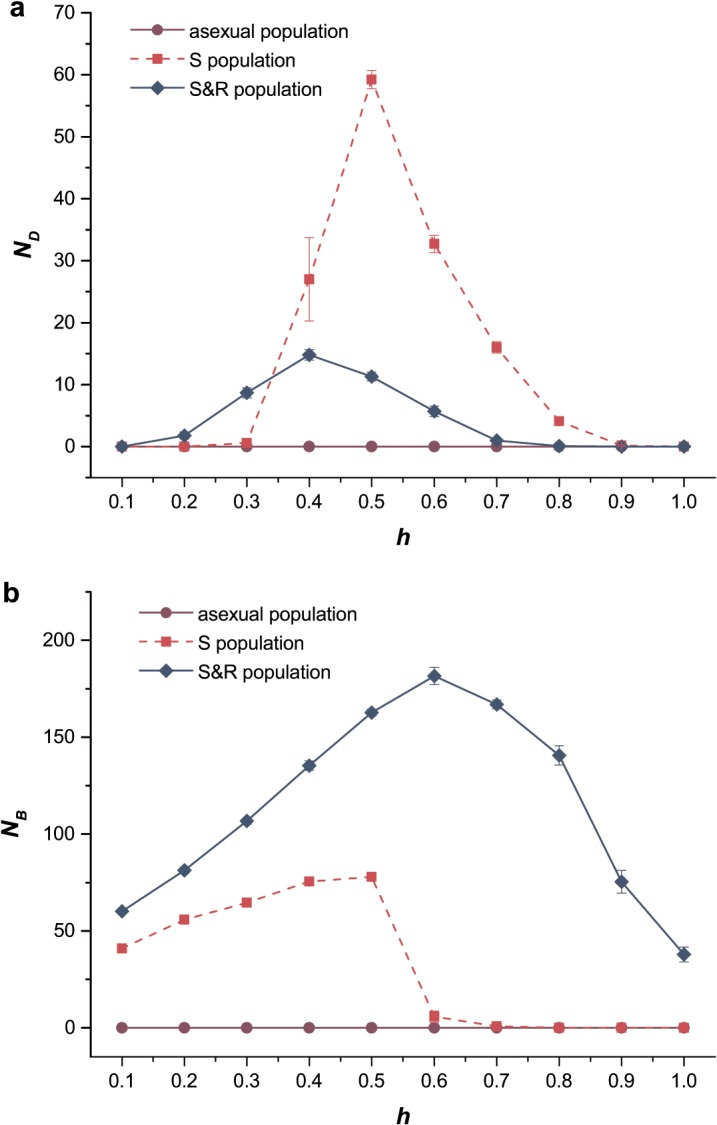
The fixation number of mutations. The fixation number of mutations in simulation as a function of the dominance coefficient *h*. Purple circles, solid lines, asexual population; red squares, dashed lines, sexual population with only segregation; blue diamonds, solid lines, sexual population with both segregation and recombination (*L* = 0.01). (a) The fixation number of deleterious mutations *N*_*D*_. *U*_*D*_ = 0.2, *s*_*D*_ = 0.05. (b) The fixation number of deleterious mutations *N*_*B*_. *U*_*B*_ = 0.002, *s*_*B*_ = 0.02. Other parameters used were the same as those in Fig 1.

The fixation number of mutations was recorded in the simulations to understand the effects of dominance on segregation and recombination. Several important tendencies were obtained from the simulation results (Fig 2). In sexual populations, the fixation number of mutations had peak values when *h* was close to 0.5. For this *h* value, segregation effectively increased the fixation number of mutations in both scenarios (exclusively deleterious mutations and exclusively beneficial mutations). The increase in fixation number of mutations was primarily due to segregation especially in the case of partially dominant deleterious mutations (or partially recessive beneficial mutations). This was because segregation increased homozygous genotypes *AA* and *aa* by converting the heterozygous genotypes *Aa*, which arose from deleterious mutations, into *AA* and *aa*. Furthermore, the average fitness of extreme genotypes (*AA* and *aa*) was higher than that of *Aa* in the above situation. In the absence of segregation in populations, the emergence of *aa* individuals must wait for a rare mutational event before a heterozygous (*Aa*) converted to a homozygous carrier. According to the research of Charlesworth and Charlesworth (1997), when *h* was less than the critical value ($h=1/(1+\sqrt{1-s})$), homozygous mutant genotypes *aa* would be subjected to higher selection pressure than heterozygous genotypes *Aa* [32]. Thus, most of the deleterious mutations were in heterozygous state *Aa*, so the fixation number of deleterious mutations would decrease with *h* (with *s*_*D*_ = 0.05 in our simulation, we have the critical value *h ≈* 0.5064).

Moreover, the S&R population had lower level fixation of deleterious mutations than S population in the presence of recombination when *h* > 0.3, since the occurrence of recombination broke the linkage between different alleles and thus weakened the hitchhiking effects [37, 38]. This indicated that recombination counteracted the effects of segregation on fixation of deleterious mutations. This conclusion was reversed for beneficial mutations, because the presence of recombination significantly increased the fixation number of mutations (Fig 2B), implying that recombination could hasten the spread of beneficial mutations and accelerate adaptive evolution [39–41]. Additionally, when mutations were partially dominant, the fixation number of deleterious mutations decreased with *h* being the stronger selection on deleterious alleles. The fixation number of beneficial mutations also decreased when *h* > 0.5. With increasing *h*, the fitness of genotypes *Aa* was close to that of the fittest genotypes *aa*. Consequently, more *A* alleles accumulated in the heterozygosity state. Thus, the weak selection on *Aa* decreased the fixation number of beneficial mutations in *a*.

Then, we focused on the mean number of mutations per chromosome (S1 Fig). The operation of Muller’s ratchet, which meant the accumulation of deleterious mutations in asexual population, would be impeded by introducing recombination and the increasing dominance coefficient [31]. Deleterious mutations, which would be eliminated by recombination, accumulated in the heterozygous state in non-recombinant populations with lower values of *h* (S1 Fig). On the other hand, both numbers of fixed beneficial mutations and aroused mutations per chromosome had a peak number with two different given values of *h*, respectively. Above this value, the fitness of heterozygous genotype *Aa* is close to that of homozygous genotype *aa*. Thus, neither segregation nor recombination could efficiently purge allele *A*, which accumulated in the heterozygous genotype *Aa*. This phenomenon led to the decrease in fixation and mean number of beneficial mutations per chromosome (Fig 2).

**Fig 3:**
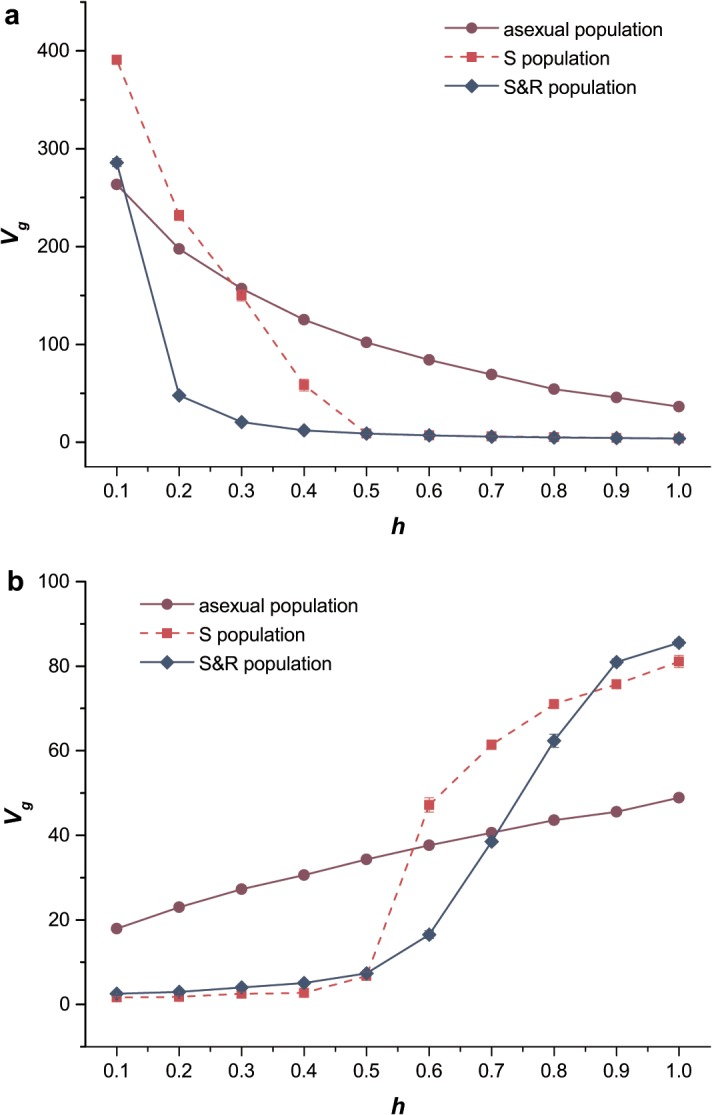
The genetic diversity of the three populations. Genetic diversity *V*_*g*_ as a function of the dominance coefficient *h*. Purple circles, solid lines, asexual population; red squares, dashed lines, sexual population with only segregation; blue diamonds, solid lines, sexual population with both segregation and recombination (*L* = 0.01). (a) Mutations were exclusively deleterious. *U*_*D*_ = 0.2, *s*_*D*_ = 0.05. (b) Mutations were exclusively beneficial. *U*_*B*_ = 0.002, *s*_*B*_ = 0.02. Other parameters used were the same as those in Fig 1.

We also investigated the effects of dominance on genetic diversity (*V*_*g*_). We compared the genetic diversity in the three populations in which mutations were exclusively deleterious and exclusively beneficial (Fig 3). When the dominance coefficient of deleterious mutations was close to 0, sexual populations had higher *V*_*g*_ than the asexual population. The same result was obtained in the case of beneficial mutations when *h* was close to 1.0. This was because in these two cases, the fitness of genotype *Aa* was close to that of the fittest genotype (*AA* or *aa*) as in Eqs (1) and (2), and it weakened the purging selection caused by segregation and recombination on the relative deleterious alleles. Therefore, the sexual populations in the presence of segregation or recombination, which could hasten the spread of mutations, had larger *V*_*g*_ than the asexual population. Meanwhile, the asexual population had a larger *V*_*g*_ than the sexual populations (S population and S&R population) in both cases where deleterious mutations were partially dominant and beneficial mutations were partially recessive. Recombination and segregation were responsible for the decrease in *V*_*g*_ as their occurrence accelerated the fixation of beneficial mutations as well as the purging of deleterious alleles, by breaking the genetic associations between alleles and loci.

### Evolution of sex modifier

**Fig 4:**
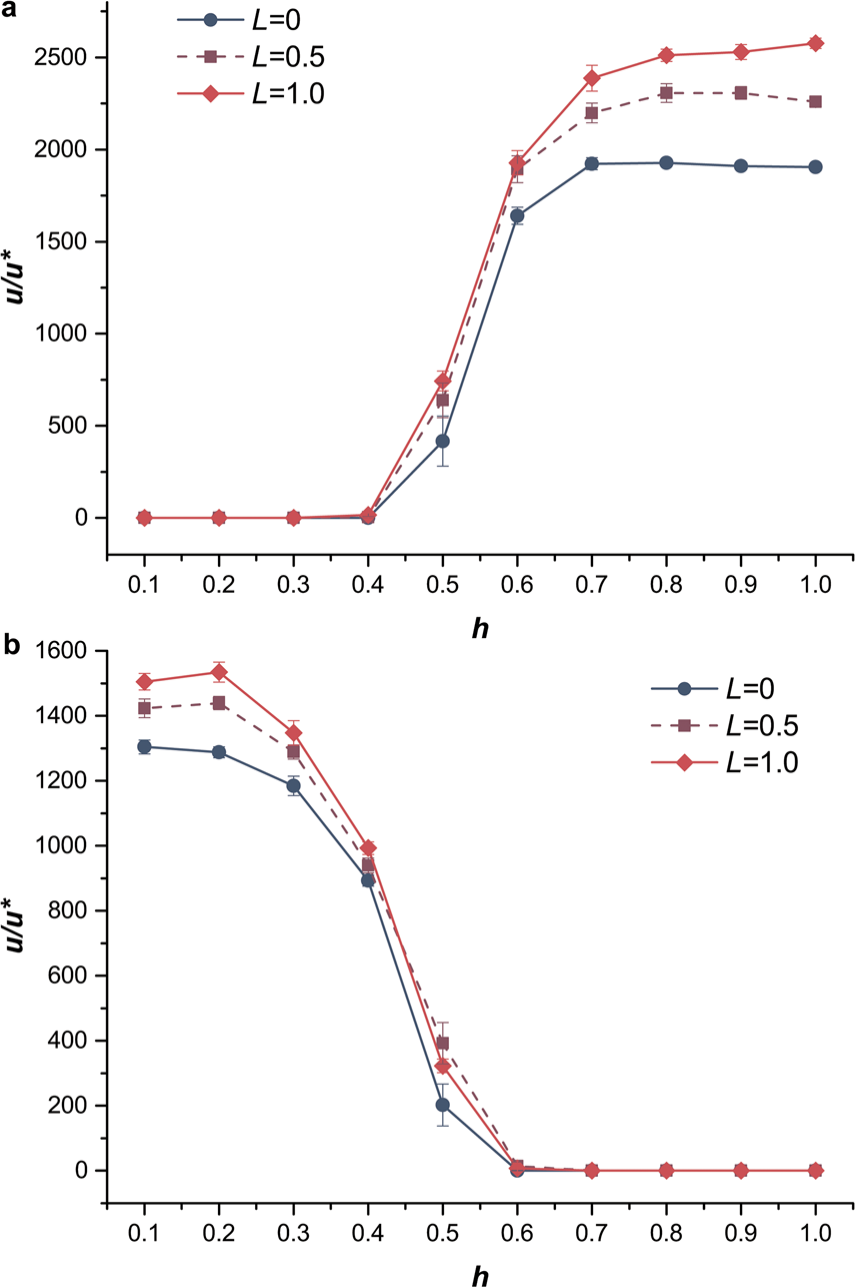
Selective advantage of a sex modifier *u/u* ^*^. Selective advantage *u/u* ^*^ as a function of the dominance coefficient *h*. Blue circles, solid lines, sexual population with *L* = 0 (only segregation); purple squares, dashed lines, sexual population with *L* = 0.5; red diamonds, solid lines, sexual population with *L* = 1.0. (a) Mutations were deleterious. *U*_*D*_ = 0.2, *s*_*D*_ = 0.05. The presence of recombination had significant effects on the relative fixation probability of the sex modifier when *h* > 0.5 (Kruskal-Wallis tests: *P*-values < 0.01). (b) Mutations were beneficial. *U*_*B*_ = 0.002, *s*_*B*_ = 0.02. The presence of recombination had significant effects on the relative fixation probability of the sex modifier when *h* < 0.5 (Kruskal-Wallis tests: *P*-values < 0.01). All statistical analyses were completed in R 3.1.0.

We tracked the fate of a sex modifier in the simulation to investigate the effects of dominance on the evolution of sex. The relative fixation probability of the sex modifier, *u/u* ^*^, was recorded in two scenarios, exclusively deleterious mutations and exclusively beneficial mutations (Fig 4), respectively, and *u/u* ^*^ > 1.0 indicated that sexual reproduction was favored [42]. First, the simulation result suggested that segregation alone (*L* = 0) obviously offered benefits to the fixation of a sex modifier in diploids, especially in cases when deleterious mutations were partially dominant or beneficial mutations were partially recessive. However, when deleterious mutations were partially recessive or beneficial mutations were partially dominant, the benefits of segregation were decreased. With extremely low or high values of *h*, respectively, this benefit decreased to 0. When deleterious mutations had a dominance coefficient *h* > 0.5, the difference of fitness between the fittest genotype *AA* (or the fittest genotype *aa* when beneficial mutations were partially recessive) and the heterozygote genotype *Aa* was higher than that in the case when deleterious mutations were partially recessive (beneficial mutations were partially dominant). The fitness of these genotypes met Eq (5). Therefore, in the two cases (deleterious mutations were partially dominant or beneficial mutations were partially recessive), segregation could efficiently eliminate deleterious alleles and drive the evolution of sex after balancing these homozygotes and heterozygotes [6]. It also means that dominant deleterious mutations and recessive beneficial mutations more likely contribute to the advantages of segregation and drive the evolution of sex. Second, the relative fixation probabilities of the sex modifier were also influenced by recombination. When deleterious mutations were partially dominant or beneficial mutations were partially recessive, the presence of recombination resulted in a significantly higher *u/u* ^*^ than that in the case of absence of recombination (*L* = 0). In the situation mentioned above, the difference in *u/u* ^*^ between the different *L* values was statistically significant (*P* < 0.05 using Kruskal–Wallis tests), which revealed that the evolution of sex favored more recombination. Noticed that in our modifier model, recombination only occurred in the mating process of individuals with sex modifiers. When deleterious mutations were partially recessive or beneficial mutations were partially dominant, the spreading speed of sex modifier was quite slow, leading to a low proportion of individuals reproduced with recombination. In this situation, the advantage of recombination could not be revealed clearly as in as in fully sexual population (Fig 1, S2 Fig).

### Effects of recombination

**Fig 5:**
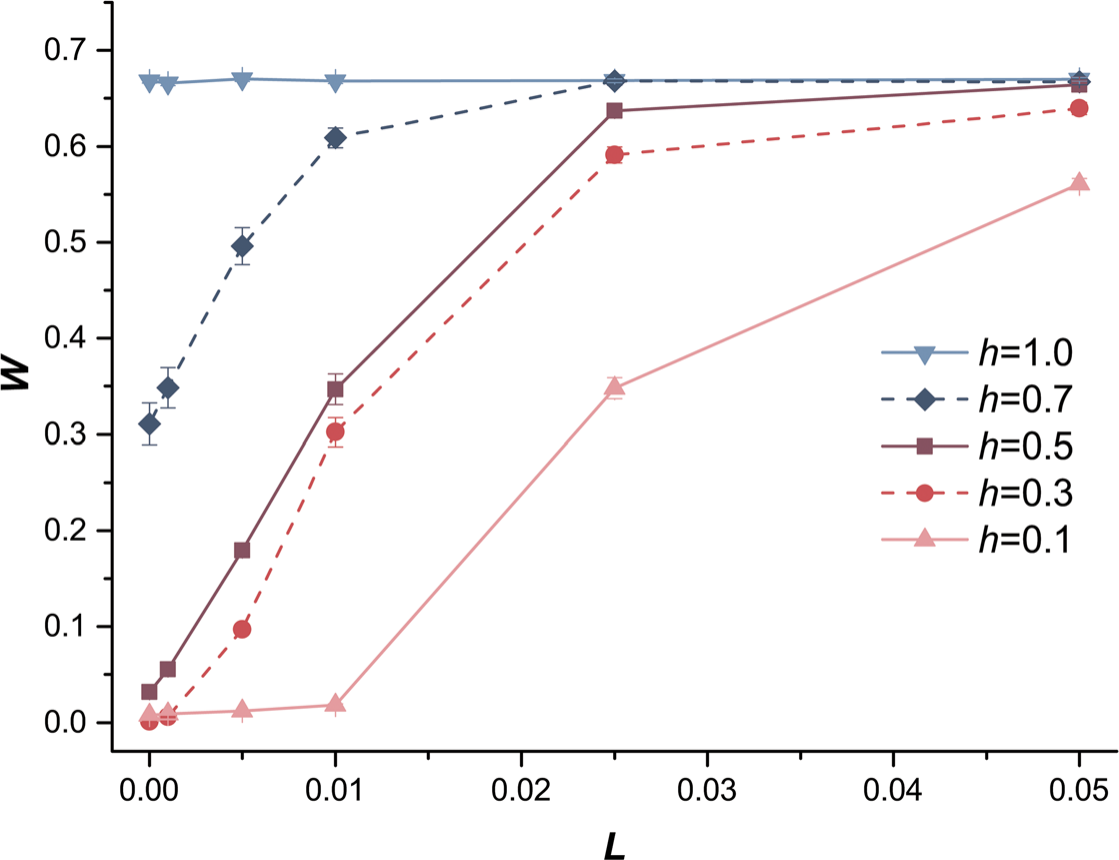
The effect of recombination on *W* under deleterious mutations. The mean fitness *W* of each population as a function of genetic map length per chromosome *L*. The deleterious mutations with dominance coefficient *h* = 0.1, *h* = 0.3, *h* = 0.5, *h* = 0.7, *h* = 1.0. Other parameters are the same in all cases: the population size *N* = 10,000, the deleterious mutation rate *U*_*D*_ = 0.2 and the strength of selection *s*_*D*_ = 0.05.

**Fig 6:**
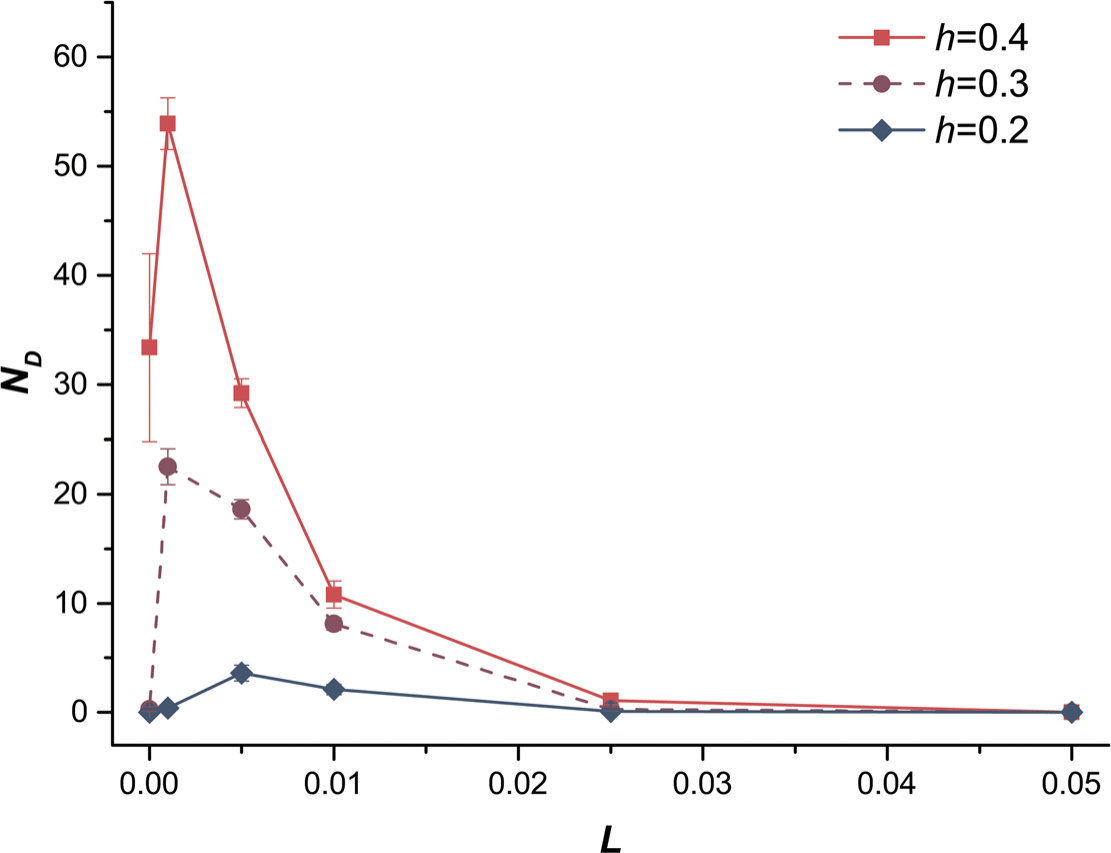
The fixation number of deleterious mutations *N*_*D*_ *vs*. genetic map length per chromosome *L*. The fixation number of deleterious mutations as a function of genetic map length per chromosome *L*. The deleterious mutations with dominance coefficient *h* < 0.5 (*h* = 0.2, *h* = 0.3, *h* = 0.4). Other parameters are the same in all cases: the population size *N* = 10,000, the deleterious mutation rate *U*_*D*_ = 0.2 and the strength of selection *s*_*D*_ = 0.05.

We also explored the effects of recombination by changing the linear genetic map length per chromosome *L*, where smaller *L* meant lower level of recombination. The mean fitness of populations with different *L* values was compared in the case of exclusively deleterious mutations (Fig 5). More recombination led to a higher *W* till the mean fitness (*W*) reached a plateau, as the effect of recombination improving the efficiency of selection had already attained the maximum. According to Fig 5, when *h* = 0.7 or 1.0, a low level (*L* > 0.01) of recombination could effectively eliminate deleterious mutations as *W* reached the plateau. Granted, a higher level of recombination was required for a small value of *h* (e.g., *h* = 0.1) to purge deleterious mutations and enable the mean fitness to approach that plateau. A low level of recombination was sufficient for higher *h* because *AA* individuals were much fitter than *Aa* individuals, and it increased the efficiency of recombination. Charlesworth (1990) studied the equilibrium properties of asexual populations, sexual populations lacking genetic recombination, and sexual populations with arbitrary recombination and suggested that the mean fitness of sexual populations increased with *L* [43]. However, the effects of *h* had not been discussed in detail. Our study revealed that the lower level of recombination could effectively increase the mean fitness of population with only deleterious mutations when the dominance coefficient was higher (e.g., *h* = 0.7 in Fig 5). We also observed that the fixation number of deleterious mutations was maximized for some intermediate values of *L*, implying that a slight recombination facilitated the fixation of deleterious mutations by hitchhiking effects when *h* < 0.5 (Fig 6). Meanwhile, a higher level of recombination effectively eliminated deleterious mutations, resulting in a decrease in the fixation number of deleterious mutations over all values of *h*. However, for beneficial mutations, both the logarithm of *W* and the fixation number of beneficial mutations increased with *L* (S2 and S3 Figs), as recombination significantly hastens the spread of beneficial mutations in diploids [39].

### Influence of population size and genetic drift

**Fig 7:**
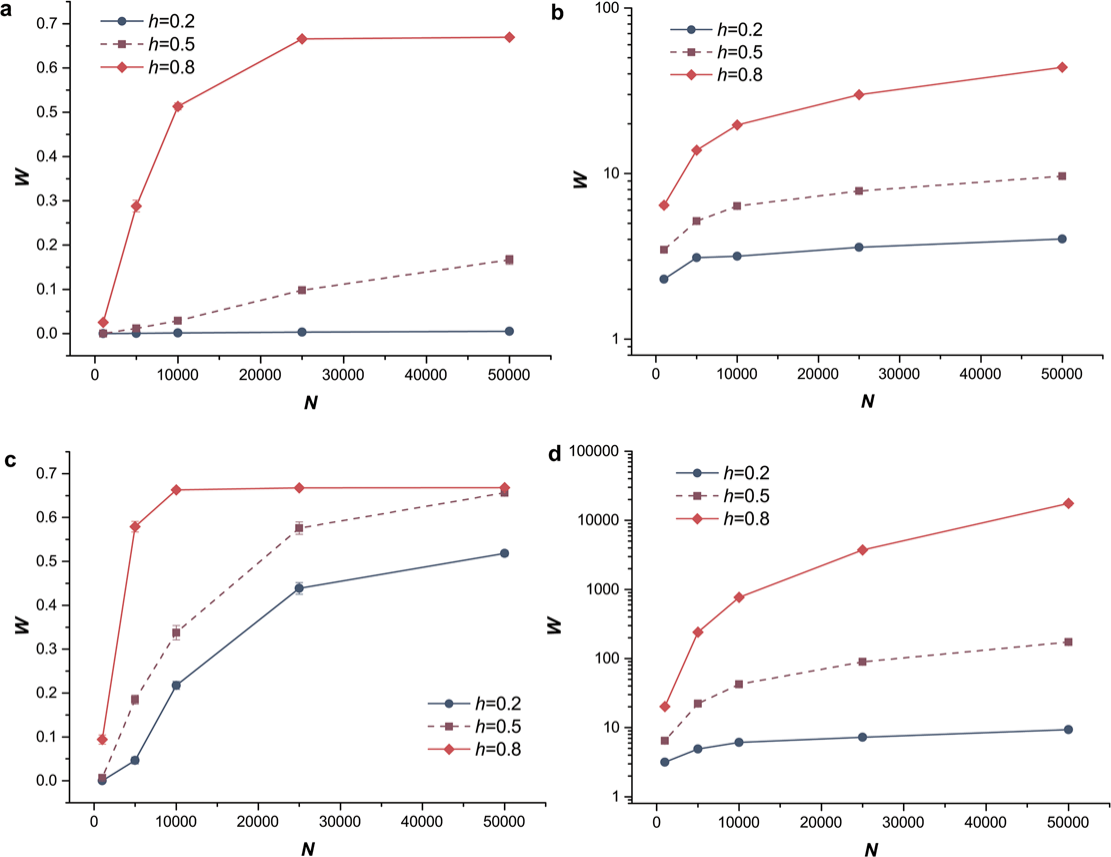
The mean fitness of populations with different sizes. Blue circles, solid lines, *h* = 0.2; purple squares, dashed lines, *h* = 0.5; red diamonds, solid lines, *h* = 0.8. The mean fitness, *W*, of each population as a function of population size *N*. (a) Sexual population with only segregation and mutations were exclusively deleterious. *U*_*D*_ = 0.2, *s*_*D*_ = 0.05. (b) Sexual population with only segregation and mutations were exclusively beneficial. *U*_*B*_ = 0.002, *s*_*B*_ = 0.02. (c) Sexual population with both segregation and recombination (*L* = 0.01). Mutations were exclusively deleterious. *U*_*D*_ = 0.2, *s*_*D*_ = 0.05. (d) Sexual population with both segregation and recombination (*L* = 0.01). Mutations were exclusively beneficial. *U*_*B*_ = 0.002, *s*_*B*_ = 0.02.

**Fig 8:**
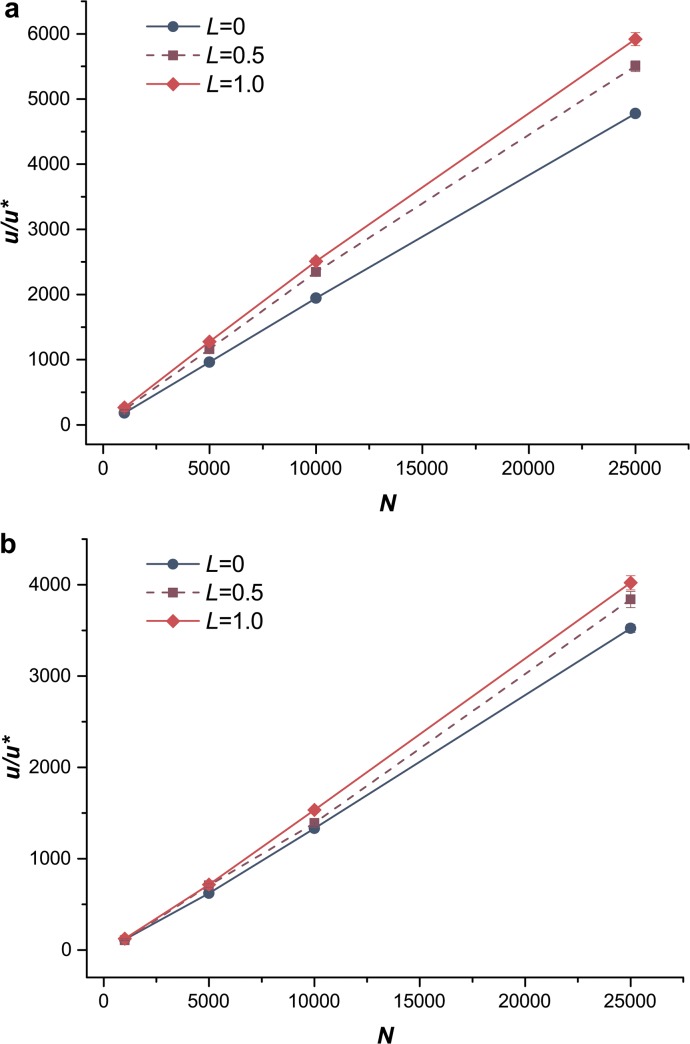
Selective advantage of a sex modifier *u/u* ^*^ with different population sizes. Selective advantage *u/u* ^*^ as a function of population size *N*. Blue circles, solid lines, sexual population with *L* = 0 (only segregation); purple squares, dashed lines, sexual population with *L* = 0.5; red diamonds, solid lines, sexual population with *L* = 1.0. (a) Mutations were exclusively deleterious. *U*_*D*_ = 0.2, *s*_*D*_ = 0.05. (b) Mutations were exclusively beneficial. *U*_*B*_ = 0.002, *s*_*B*_ = 0.02.

As genetic drift is one of the basic mechanisms of evolution, it was considered in our study by changing the population size, which described the changes in allele frequencies due to genetic drift [44]. Our simulation indicated that the mean fitness *W* of population increased with population size *N* (Fig 7). The genetic drift is stronger for smaller populations. As a result of small population size, more deleterious mutations became fixed in the simulation (both for the S and S&R populations). So, larger population size reduced the influence of genetic drift and led to higher mean fitness. For beneficial mutations scenarios, if the population size is small, then genetic drift hinders the fixation of even highly beneficial mutations [11]. Meanwhile, populations with dominant beneficial mutations have higher mean fitness because the stronger selection on dominant mutations weakens the influences of genetic drift. At the same time, we also explored the effects of changing population size (*N*) undergoing genetic drift on the relative fixation probability of a sex modifier, *u/u* ^*^. An increasing *N* significantly increased the fixation probability of the sex modifier (Fig 8). This result indicated that sexual reproduction would be more adaptive for larger populations with weak genetic drift; in other words, increasing population size provides substantial benefits to the evolution of sex.

## Discussion

Most eukaryotes reproduce sexually; however, sexual reproduction often seems more troublesome than it is worth. For example, much energy is spent for finding willing mates for sex. Therefore, the origin and maintenance of sex is one of the important issues in evolutionary biology. This problem extends even further because of the facts that species are haploid or diploid and gametes are generated by meiosis. In diploid species, the process of meiosis involves recombination and segregation. As we know, dominance is an important factor, which is closely associated with segregation in diploids and affects its contribution to the advantages of sex. However, how dominance affects the contribution of segregation has been less studied. Our research focuses on the influence of dominance on segregation, particularly on the relative advantages of segregation and recombination to sex in diploid populations. In brief, it was observed that sexual reproduction would be more adaptive in cases of dominant deleterious mutations and recessive beneficial mutations. This result indicates that different degrees of dominance could make a diverse contribution to the advantage of sex. That is to say, a certain degree of dominance will lead a sexual population to be more adaptive to the environment. When sex occasionally appeared in an asexual population, it was not easy for these ‘sex-related genes’ to spread and get fixed, especially in a fluctuating environment. But the emergence of dominance could solve this problem. According to our result, sexual individuals are more adaptive in this fluctuating environment under the driving of dominant deleterious mutations and recessive beneficial mutations. Therefore, sex has a higher probability to get fixed. Overall, our research indicates that dominance plays a more important role than what we believe in the evolution of sex and improves our understanding of the origin of sex.

We adopted computer simulations to investigate the relative effects of recombination and segregation in diploids and how the dominance values affect the selective advantage of sexual reproduction. It is well known that most mutant genes are partially recessive [45–47], but the effect of dominance on segregation and evolution of sex remains unclear. Studies have shown that the effects of dominance on population evolution may be related to the maintenance of sexual reproduction [29, 48–50]. Otto (2003) investigated the effects of dominance on sex modifiers and found that inbreeding could increase the advantage of sex in the presence of dominant deleterious mutations. His study also showed that a higher value of *h* favors sex modifiers [50]. Meanwhile, previous studies revealed that, for exclusively deleterious mutations, the frequency of mutated alleles under multiplicative selection depended on *h* [26], and the rate of sex evolved toward relatively high values when *h* ≥ 0.3 [29]. However, all these studies were about deleterious mutations. Using a sex modifier diploid model, we investigated the relative effect of segregation and recombination for both deleterious and beneficial mutations. Our simulation suggested that segregation more commonly had a major impact in cases when mutations were deleterious and partially dominant or beneficial and partially recessive. We also found that the benefits of segregation alone could overcome the cost of sex and drive the evolution of sex as long as deleterious mutations were partially dominant or beneficial mutations were partially recessive.

Moreover, we investigated the effects of recombination by varying the genetic map length per chromosome and found that recombination could effectively increase the mean fitness of populations and hasten the spread of beneficial mutations, which is in agreement with other results [39, 43]. The introduction of recombination could markedly increase the mean fitness and the fixation number of beneficial mutations compared with the case absence of recombination (Figs 1B and 2B), thereby accelerating the adaptive evolution of populations. In other words, recombination improved the efficiency of selection by purging deleterious alleles. Thus, the relative contribution of recombination to selection increased with higher *L*. When deleterious mutations were partially recessive, recombination became the major factor that increased the mean fitness of the sexual population (Fig 1A).

In conclusion, the dominance of mutations had a strong influence on the benefits of segregation. When deleterious mutations were partially dominant or beneficial mutations were partially recessive, segregation had a relatively significant effect, which could explain the evolution of sex in diploids alone (Fig 4). With low values of *h* for deleterious mutations or high values of *h* for beneficial mutations, segregation did not facilitate the sexual reproduction in diploids.

Besides, some other studies also explored the advantage of sex with the use of experiments on yeast and *Aspergillus nidulans* populations. These studies revealed that sex could increase the rate of adaptation to harsh environments and slow down the accumulation of deleterious mutations [51, 52]. In another study on the fixation of beneficial mutations in *Chlamydomonas reinhardtii*, the advantage of sex is noticeable with large population size but insignificant in small populations [53]. As experiments might have some limits in variable control, simulations can give us results when some factors are not experimentally measurable. Therefore, it would be better to combine laboratory experiments with computer simulations to investigate the influences of other important factors on the advantage of sex in future studies.

In our simulation, asexual population was assumed without recombination in order to make direct comparison with sexual populations in the presence or absence of recombination. However, genetic recombination is ubiquitous among species [54, 55]. The simultaneous occurrence of beneficial mutations and deleterious mutations was excluded in our simulations, but it should be studied in the future to understand the mechanism of sex evolution. To achieve this purpose, additional theoretical models are required. Meanwhile, it may be interesting to consider other important factors such as inbreeding and epistasis.

## Supporting Information

S1 Fig Mean number of mutations per chromosome. The mean number of mutations per chromosome as a function of the dominance coefficient *h*. Purple circles, solid lines, asexual population; red squares, dashed lines, sexual population with only segregation; blue diamonds, solid lines, sexual population with both segregation and recombination (*L* = 0.01) (a) The mutations were exclusively deleterious. *U*_*D*_ = 0.2, *s*_*D*_ = 0.05. (b) The mutations were exclusively beneficial. *U*_*B*_ = 0.002, *s*_*B*_ = 0.02. The population size *N* = 10,000.
(EPS)

S2 Fig The effect of recombination on *W* under beneficial mutations. The logarithm *W* of each population as a function of dominance coefficient *h*. The genetic map length per chromosome *L* was set to 0, 0.01, 0.1, 0.5 and 1.0. Other parameters were the same in all cases: the population size *N* = 10,000, the beneficial mutation rate *U*_*B*_ = 0.002 and the strength of selection *s*_*B*_ = 0.02.
(EPS)

S3 Fig The fixation number of beneficial mutations *N*_*B*_ *vs*. dominance coefficient *h*. The fixation number of beneficial mutations *N*_*B*_ as a function of the dominance coefficient *h* for cases with genetic map length per chromosome *L* was set to 0, 0.01, 0.1, 0.5 and 1.0. Other parameters used: the population size *N* = 10, 000, the mutation rate *U*_*B*_ = 0.002 and the strength of selection *s*_*B*_ = 0.02.
(EPS)
